# Spontaneous Atraumatic Extensor Pollicis Longus Rupture in the Nonrheumatoid Population

**Published:** 2013-02-28

**Authors:** Erin M. Rada, Sachin M. Shridharani, Scott D. Lifchez

**Affiliations:** Department of Plastic and Reconstructive Surgery, Johns Hopkins University, Baltimore, MD

## Abstract

**Introduction:** Extensor pollicis longus (EPL) tendon rupture is a well-described phenomenon in patients with rheumatoid arthritis. Mechanisms of EPL tendon rupture in the nonrheumatoid population have also been described and include traumatic rupture, repetitive motion strain, and steroid injection into the tendon. **Methods:** The operative records for patients undergoing extensor pollicis longus reconstruction by the senior author were reviewed. Patients with a history of trauma to the wrist or inflammatory arthropathy were excluded. **Results:** We identified 3 patients who presented with spontaneous EPL tendon rupture. These patients reported no risk factors (as listed earlier) or inciting event. All 3 patients had some exposure to local steroids but this exposure was not at the site of subsequent tendon rupture. All patients were operatively repaired and went on to full recovery of EPL function. **Discussion:** In patients with sudden loss of extension of the thumb interphalangeal joint, a thorough history of steroid exposure including local steroid exposure remote to the affected EPL tendon may be relevant.

Extensor tendon rupture is frequently observed in patients with rheumatoid arthritis. Inflammatory synovium and rough bone edges due to erosions both contribute to this process.^1^

In the nonrheumatoid population, multiple etiologies can cause extensor tendon rupture. In this group, rupture is primarily traumatic and of 2 main etiologies. Trauma may produce enough mechanical stress to the tendon to cause spontaneous rupture.^2^^-^4 Fracture may cause gradual wear of the extensor pollicis longus (EPL) tendon either from the fracture edges or from internal fixation hardware used to stabilize the fracture.^5^ Rupture in these patients may be due to mechanical wearing of the tendon fibers and/or disruption of the mesotenon thereby disrupting vascular supply.^2^^,^^6^ The tendon becomes weakened and more susceptible to rupture. Tophaceous gout and osteoarthritis can cause EPL wear and rupture via a similar mechanism.^7^^-^9 Case reports have also described EPL rupture attributed to repetitive motion strain as in skiers, goalkeepers, and other athletes.^10^^-^11

Corticosteroids have been implicated in tendon rupture in multiple areas of the body. Systemic or local steroid use (at the site of rupture) has also been implicated in extensor tendon rupture. Lee first reported rupture of the Achilles tendon after hydrocortisone.^12^ Achilles rupture has also been reported in association with systemic corticosteroid use.^13^ Björkman and Jörgsholm^5^ reported 8 patients with EPL rupture in association with steroid use. In all of these patients, steroids were administered systemically or injected into the tendon that ultimately ruptured.

## METHODS

The charts the senior author's patients who underwent reconstruction of the EPL were reviewed. We identified 3 patients who underwent reconstruction for spontaneous tendon rupture with no history of trauma or inflammatory disorder. Patient history and preoperative imaging along with operative findings were reviewed to identify any risks or exposures common to all 3 patients. All 3 patients had exposure to corticosteroids in the ipsilateral extremity of eventual EPL rupture.

## RESULTS

### Patient 1

A 46-year-old right-hand-dominant man was referred for evaluation of painful catching of the right thumb for 3 months. The patient denied any history of trauma to the hand. On physical examination, he had a nodule in the flexor policis longus (FPL) and triggering. There was no pathology on X-rays. One milliliter of 1:1 solution of triamcinolone (40 mg/mL) and bupivacaine 0.5% was injected into the patient's right thumb flexor tendon sheath at the level of metacarpal head. Three months later, he underwent a second flexor sheath injection for symptoms that had recurred 3 weeks prior. He did not get any relief from the second injection and was scheduled for A1 pulley release.

On the day of surgery, he noted a recent sudden onset of the inability to actively extend the thumb interphalangeal (IP) joint. He was tender over the anatomic snuffbox. He again denied any recent trauma. Physical examination confirmed loss of active thumb IP extension with no tenodesis effect and no evidence of scars or laceration on the hand. In the operating room, the patient's A1 pulley was released. The EPL was explored from mid-metacarpal to extensor retinaculum, and tendon rupture was noted over the carpus. Primary repair was performed. In the ensuing 3-month period, the patient recovered full IP joint (IPJ) active extension.

### Patient 2

A 54-year-old right-hand-dominant woman presented with spontaneous loss of extension of the right thumb at the IPJ for the preceding 5 days. The patient denied any trauma to the hand. She stated that she was carrying less than 10 pounds of supplies at work when she felt a “pop” and discomfort in the distal dorsal forearm. The patient works as a physical therapist and performs dexamethasone and hydrocortisone iontophoresis. Her physical examination was notable for the absence of right thumb IPJ active extension. Magnetic resonance imaging (MRI) showed rupture of the EPL tendon, as well as evidence of synovitis at the third and fourth compartments of the wrist.

**Figure 1:**
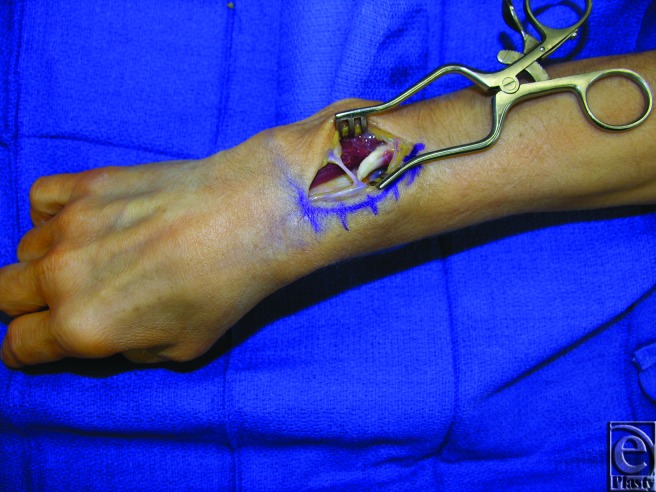
Intraoperative photograph of patient 2 demonstrating complete rupture of the right EPL tendon with proximal end in visible in the dorsal forearm. EPL indicates extensor pollicis longus.

**Figure 2:**
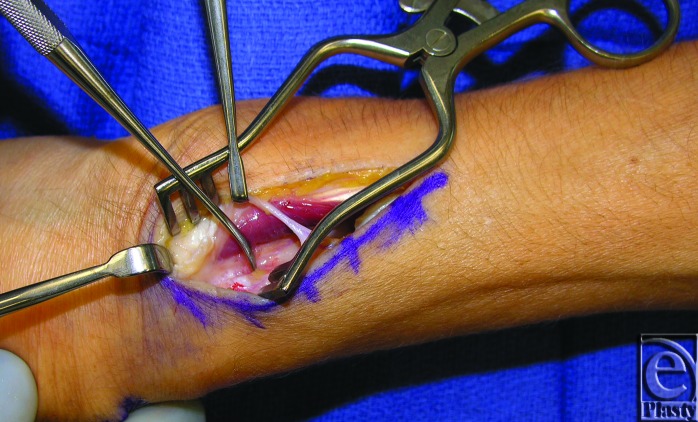
Intraoperative photograph of patient 2 with a Freer elevator identifying Lister's tubercle.

In the operating room, the patient had a ruptured EPL tendon with the proximal end in the mid-forearm and the distal end in the anatomic snuffbox (Fig 1). Repair was performed with an ipsilateral palmaris longus tendon graft. The synovitis noted was resected. There was no obvious bony abnormality or rough edges of Lister's tubercle; however, the third compartment turned at a sharp angle at the distal end of Lister's tubercle (Fig 2).

At the patient's 1-month follow-up, she stated she was doing well except that she had pain in her left dorsal thumb proximally. On examination, she had normal motion of the left thumb IP and no crepitus or pain at the left third compartment of the wrist. Given her history of right EPL rupture without pain, we obtained an MRI that showed first through third compartment synovitis. Given the patient's history of right EPL rupture, left EPL synovectomy and transfer out of third compartment was recommended.

**Figure 3:**
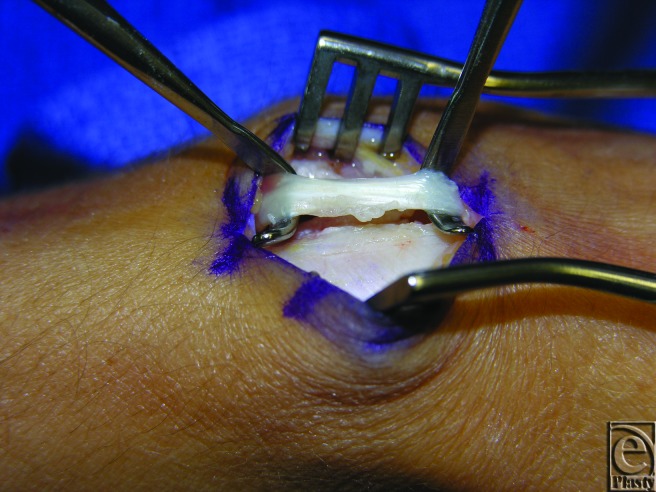
Intraoperative photograph of patient 2 with visible fraying of left EPL tendon. EPL indicates extensor pollicis longus.

In the operating room, the EPL tendon was examined within the third dorsal compartment. There was significant fraying of the tendon (<50%) along its radial aspect where it was in contact with Lister's tubercle (Fig 3). Lister's tubercle was inspected, and no injury or rough edge was noted. The tendon was completely released and transposed radially. Synovium was removed and sent to pathology and microbiology for further evaluation.

In the ensuing 3-month period, the patient underwent splinting and hand therapy. Her recovery was uneventful. For both surgeries, the resected synovium showed mild chronic inflammation; all cultures were negative for growth.

### Patient 3

A 47-year-old right-hand-dominant man presented with pain on the dorsum of his left wrist and lateral elbow. The patient denied any past trauma to the hand and works as a machinery operator. Physical examination showed tenderness to palpation at the intersection of the tendons of the left first and second dorsal compartments. There was a separate locus of pain at the left lateral epicondyle of the humerus. We injected 40 mg each of triamcinolone into the intersection of the tendons of the left first and second dorsal compartment and into the left lateral epicondyle of the humerus. Symptoms were relieved for 8 months, at which time the patient returned to clinic and we injected both areas a second time. Seven months after this visit, he returned with recurrent lateral elbow symptoms.

**Figure 4:**
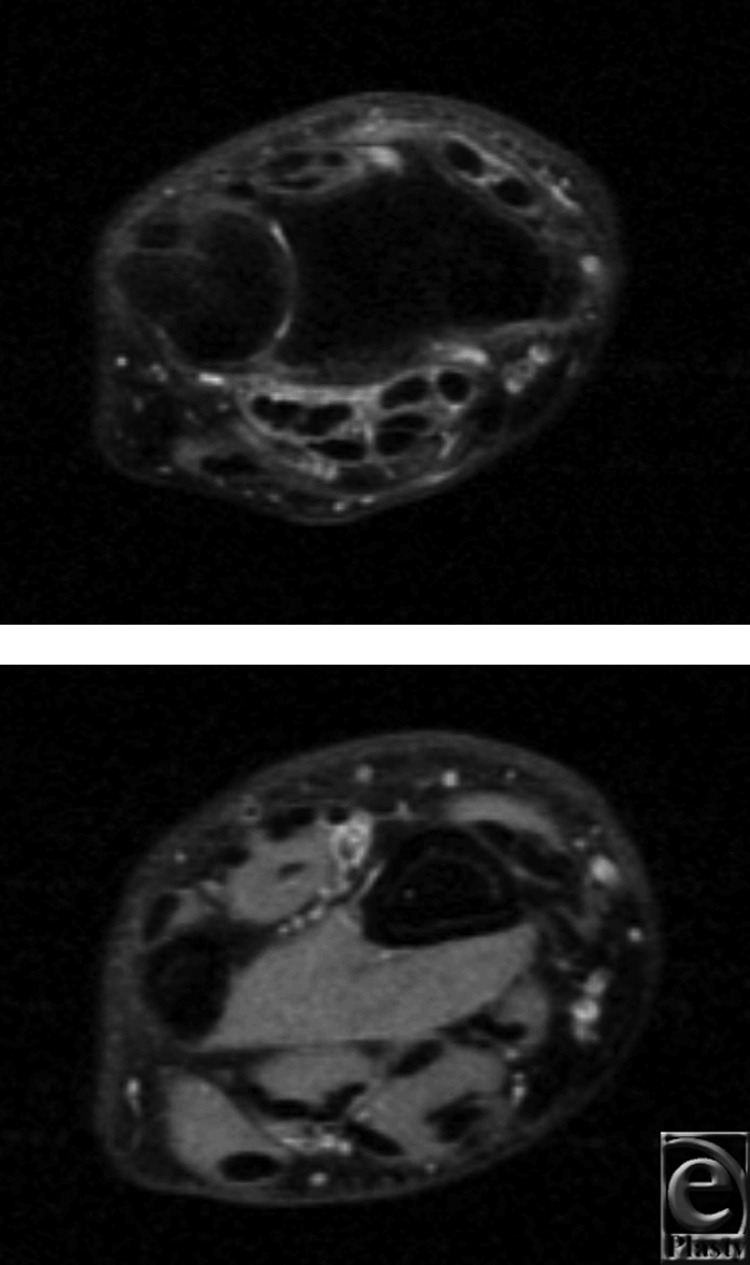
T2-weighted MRI images of patient 3 demonstrate inflammation and the absence of a tendon within the third dorsal compartment. MRI indicates magnetic resonance imaging.

Two months later, he underwent left lateral epicondylectomy with suture-repair of the extensor digitorum communis to the extensor carpi radialis longus. At his 1-month follow-up, the patient had a new onset left dorsal wrist pain and the inability to extend the thumb IPJ. Magnetic resonance imaging confirmed rupture of the EPL tendon at the third compartment (Fig 4). The patient was taken to the operating room where EPL reconstruction with a palmaris tendon graft was performed. At the patient's 1-month follow-up, elbow pain had resolved and full EPL function returned.

## DISCUSSION

Tendon rupture, particularly EPL tendon rupture, has been described in association with a variety of associated factors—rheumatoid arthritis, prominent bony anatomy or hardware, blunt trauma, and repetitive strain. In this case series, we report 3 patients who presented with complete extensor tendon rupture, one of whom also had a partial EPL tendon rupture on the contralateral wrist. Although repetitive strain has been implicated in EPL rupture in several case reports,^10^^-^11 it was not contributing factor in our series. Bony prominences, both congenital and acquired, are also reported as a potential cause of extremity tendon rupture.^2^^,^^5^^,^^6^ None of the patients in this report had any evidence of any anatomical predisposition for tendon rupture on imaging, physical examination, or inspection at the time of operation.

Since the first reports of tendon rupture in association with steroid use appeared, multiple studies have investigated the mechanism by which steroid may cause tendon rupture. Triamcinolone suppresses proteoglycan production by human tenocytes.^14^ This may negatively affect the viscoelastic properties of the tendon. Triamcinolone also suppresses human tenocyte cellular activity and collagen production, which may disrupt the structure of the tendon.^15^ Both of these factors may promote spontaneous tendon rupture.

All 3 patients in this report had steroid exposure in the ipsilateral extremity. Patient 1 received two steroid injections into the thumb flexor tendon sheath. Patient 2 did not have a steroid injection to the affected extremities, but routinely performs dexamethasone and hydrocortisone iontophoresis as a physical therapist and notes that occasionally the steroid solution would contact her skin proximal to her glove when positioning a patient for treatment. Patient 3 received 2 steroid injections into both the intersection of the tendons of the left first and second dorsal compartment and into the left lateral epicondyle of the humerus. The amount of steroid that each patient received was low with minimal systemic absorption. Furthermore, the sites of steroid exposure, while on the affected extremity, were all 4 cm or greater away from the location of subsequent EPL tendon rupture.

This case series suggests that steroid injection in a neighboring region may also be associated with EPL rupture. While steroid injection directly into the third dorsal compartment and systemic steroid use has been implicated in tendon rupture,^16^ these were not factors in our patients. We considered technical error as a possible explanation for these findings. Given the distance of the EPL rupture from the site of injection and that all injections were performed by the senior author, we consider this unlikely. Pauwels and colleagues^17^ demonstrated in an equine model that triamcinolone injected at the distal interphalangeal (DIP) joint achieved therapeutic concentrations at the navicular bursa.

In the absence of anatomical variation or an inciting event, diagnosis of EPL rupture may be lower on the examining physician's differential. The current study suggests that a regional exposure to steroid in the ipsilateral extremity is associated with tendon rupture. Magnetic resonance imaging or ultrasonography can be helpful to confirm the diagnosis and localize the rupture. Further study is necessary to better determine whether corticosteroids are causative of tendon rupture in these circumstances, and if so, by what mechanism.
